# High *KRT8* Expression Independently Predicts Poor Prognosis for Lung Adenocarcinoma Patients

**DOI:** 10.3390/genes10010036

**Published:** 2019-01-10

**Authors:** Longxiang Xie, Yifang Dang, Jinshuai Guo, Xiaoxiao Sun, Tiantian Xie, Lu Zhang, Zhongyi Yan, Hamel Amin, Xiangqian Guo

**Affiliations:** 1 Cell Signal Transduction Laboratory, Bioinformatics Center, Department of Preventive Medicine, Institute of Biomedical Informatics, School of Basic Medical Sciences, Henan University, Kaifeng 475004, China; xielongxiang123@126.com (L.X.); 15736875532@139.com (Y.D.); 15890303356@139.com (J.G.); 15736871303@139.com (X.S.); 15206668032@139.com (T.X.); 13783992847@139.com (L.Z.); 13526863491@139.com (Z.Y.); 2 Public Health Research Institute at New Jersey Medical School, Rutgers State University of New Jersey, 225 Warren Street, Newark, NJ 07103, USA; hamelamin@gmail.com

**Keywords:** KRT8, LUAD, LUSC, prognostic marker

## Abstract

Keratin 8 (KRT8), a type II basic intermediate filament (IF) protein, is essential for the development and metastasis of various cancers. In this study, by analyzing RNA-seq data from the Cancer Genome Atlas (TCGA)-lung adenocarcinoma (LUAD) and lung squamous cell carcinoma (LUSC), we have determined the expression profile of *KRT8*, and assessed its prognostic significance and the possible mechanism underlying the dysregulation. Our results showed that *KRT8* mRNA expression was significantly up-regulated in both LUAD and LUSC tissues compared with normal lung tissues. The high *KRT8* expression group for LUAD patients significantly reduced overall survival (OS) and recurrence-free survival (RFS). Univariate and multivariate analysis revealed that *KRT8* expression was an independent prognostic indicator for poor OS and RFS in LUAD patients. However, *KRT8* expression had no prognostic value in terms of OS and RFS for LUSC. By exploring DNA copy number alterations (CNAs) of the *KRT8* gene in LUAD, we found that DNA low copy gain (+1 and +2) was associated with elevated *KRT8* mRNA expression. From the above findings, we have deduced that *KRT8* is aberrantly expressed in LUAD tissues and that its expression might independently predict poor OS and RFS for LUAD patients, but not for LUSC patients.

## 1. Introduction

Keratins (KRTs) predominantly expressed in epithelial tissues can be divided into two types—I (acidic forms) and II (basic forms) of intermediate filament (IF) proteins—based on their biochemical characteristics [1,2]. KRTs contain 54 identified members, that is, 28 of type I (K9–K28) and 26 of type II (K1–K8 and K71–K74) [3]. KRTs comprising of a majority of intermediate filament (IF) are the key components of cytoskeleton [4] and are involved in many cellular processes, including mitosis, differentiation, and apoptosis [3]. For instance, Li et al. have demonstrated that overexpression of KRT13 directly promotes primary prostate cancer to metastasize to bone and brain tissue [5]. Escobar-Hoyos et al. have found that overexpression of KRT17 is identified as a novel unfavorable diagnostic and prognostic biomarker with regard to cervical cancer [6]. Recently, Liu et al. have demonstrated that a significant upregulation of KRT17 in lung adenocarcinoma (LUAD) tissues can promote cell proliferation and invasion, and its high expression is associated with poor overall survival in LUAD patients [7]. In addition, van Sprundel et al. have shown that KRT19 expression is correlated with poor differentiated histology and more aggressive behavior in hepatocellular carcinoma (HCC) [8]. 

Previous studies have shown that *KRT8* expression is significantly elevated in various human cancers, including bladder cancer [9], breast cancer [10], kidney cancer [11], pancreatic cancer [12], and lung cancer [13]. *KRT8* mRNA and protein expression has been found to be upregulated in gastric cancer (GC) tissues, and its high expression has been observed to promote the cell progression and metastasis of GC cells and produce unfavorable outcomes for patients with GC [14]. KRT8 upregulated expression can promote the metastasis of clear cell renal cell carcinoma (ccRCC) cells by up-regulating IL-11 expression, inducing IL-11 autocrine, and initiating the STAT3 signaling pathway [15]. Loss of keratin 8/18 can regulate oncogenic potential by controlling various signaling pathways, including TMS1-NF-κB signaling and MARCKSL1-Paxillin1-Rac axis, in skin squamous cell carcinomas (SCC) [16]. The KRT8 protein can bind to annexin A2 and mediate both the apoptosis and the redox pathway in anaplastic thyroid carcinoma (ATC) [17]. KRT8 overexpression mediates resistance to cadmium-induced adaptation and carcinogenesis [18]. In some cancers, upregulation of KRT8 has been considered a valuable prognostic marker, including for gastric cancer [14], oral squamous cell carcinomas [19], and ccRCC [15]. It has also been considered a promising indicator for differentiating between the diagnoses of leukoplakia [20] and head-and-neck carcinomas (HNC) [21]. A recent study has shown that a high KRT8/18 ratio correlates with an aggressive HCC phenotype and can be treated as a novel biomarker for HCC patients [22]. However, little is known regarding the effects of *KRT8* on the pathological processes of lung adenocarcinoma (LUAD) and the prognostic values associated with its expression.

In this study, by using large sequencing data with patient information from TCGA, we aimed to examine the expression profile of *KRT8* in LUAD and LUSC, and to investigate its prognostic significance in these subtypes. 

## 2. Materials and Methods

### 2.1. Retrospective Analysis Using TCGA Data

This study is a retrospective study which used TCGA level 3 data, with access provided by the University of California, Santa Cruz (UCSC) Xena browser (https://xenabrowser.net/) [23]. Molecular, clinicopathological, and more than 10 years’ survival data of over 1000 TCGA_LUAD and LUSC patients were recorded. Primary tumor tissues from 514 LUAD patients and 501 LUSC patients were collected for RNA-seq. 502 of the 514 LUAD patients and 494 of the 501 LUSC patients had complete survival data. Clinicopathological parameters of LUAD and LUSC patients with primary tumors, including age at diagnosis, gender, smoking history, pathologic stage, living status, recurrence-free survival (RFS), and OS were downloaded for survival-curve analysis. Kaplan-Meier curves of OS and RFS were mapped to assess the survival difference between patients grouped with high or low *KRT8* expression. The gene level thresholded genomic identification of significant targets in cancer 2 (GISTIC2)-processed copy number alterations (CNAs) data of LUAD were also downloaded and examined using the UCSC Xena browser.

### 2.2. Immunohistochemistry (IHC) Staining

Immunohistochemistry images of KRT8 protein expression in normal lung tissues and lung cancer tissues including LUAD and LUSC were downloaded and examined from the Human Protein Atlas (HPA) (http://www.proteinatlas.org/) [24].

### 2.3. Kaplan-Meier Plotter Data Mining Analysis

The association between the prognostic value of *KRT8* and OS in 720 LUAD patients and in 524 LUSC patients was examined by data-mining an online survival analysis software Kaplan-Meier plotter using transcriptomic data [25]. The patients were divided into two groups by using the 50% cut-off value for *KRT8* expression; the hazard ratio (HR) with 95% CI and log-rank *p*-value were directly calculated and generated using the website.

### 2.4. Oncomine Data-Mining Analysis

Oncomine (https://www.oncomine.org/resource/main.html) is a cancer microarray database and data-mining platform which can help researchers easily acquire gene expression information for human cancer tissues and cells [26]. The selected parameters for inclusion of studies, including *p*-value < 0.05, fold change ≥ 2, and gene ranking in the top 10%, were set up in this database.

### 2.5. Data-Mining Analysis of Gene Expression Omnibus (GEO) Database

Treatment-related transcriptome microarray datasets with accession numbers GSE21656 [27], GSE6400 [28], and GSE6914 [29] were downloaded from the GEO database, and normalized raw transcriptome data were reanalyzed to assess the effect of *KRT8* mRNA expression on responses to chemotherapy.

### 2.6. Statistical Analysis

SPSS 19.0 (SPSS Inc., Chicago, IL, USA) and GraphPad Prism 5.0 (GraphPad Inc., La Jolla, CA, USA) software were used for statistical analysis. χ^2^ tests were conducted to explore the correlation between *KRT8* expression and clinicopathological factors. The mRNA expression of *KRT8* in LUAD and LUSC cancer tissues was compared with that in normal tissues, using a Students’ *t*-test to calculate a *p* value. Kaplan-Meier curves of OS and RFS (using TCGA-LUAD data) were mapped by GraphPad Prism 5.0 by setting the median *KRT8* expression as the cut-off. A log-rank test was performed to examine the significant differences between the survival curves of patients. Univariate and multivariate Cox regression models were performed to assess the prognostic role of *KRT8* in terms of OS and RFS for LUAD and LUSC patients using SPSS. Risk factors (*p* < 0.2) analyzed by univariate analysis were selected for multivariate Cox regression analysis using a stepwise regression method. A value of *p* < 0.05 was considered statistically significant.

## 3. Results

### 3.1. Both LUAD & LUSC Tissues Had Significantly Increased KRT8 Expression Compared with Normal Tissues

**Figure 1:**
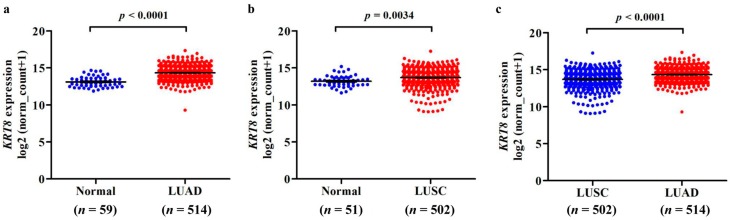
*KRT8* mRNA expression was up-regulated in both lung adenocarcinoma (LUAD) and lung squamous cell carcinoma (LUSC) in comparison with normal lung tissues. (**a**,**b**) Plots charts of *KRT8* expression in LUAD, LUSC, and their corresponding normal lung tissues. (**c**) Comparison of *KRT8* expression in LUAD and LUSC tissues.

Using TCGA RNA-seq data, we first analyzed *KRT8* mRNA expression in both lung cancer tissues and normal tissues. Results showed significantly elevated *KRT8* expression in both LUAD (*n* = 514) and LUSC (*n* = 502) tissues compared with the normal controls (Figure S1 and Figure 1a–b). In addition, *KRT8* expression in LUAD was higher than that in LUSC (Figure 1c). 

**Figure 2:**
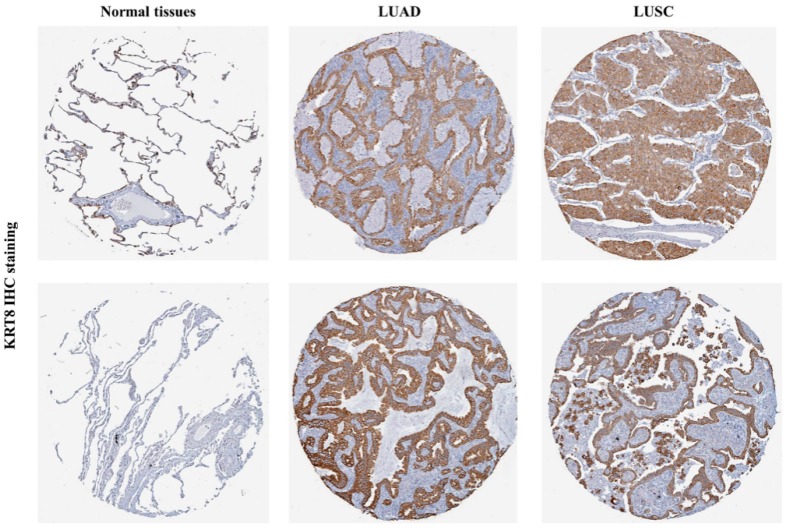
KRT8 protein expression was significantly higher in both LUAD and LUSC tissues in comparison with normal respiratory epithelial tissues. KRT8 immunohistochemistry (IHC) staining images: in normal respiratory epithelial tissues (**left**), in LUAD tissues (**middle**), and in LUSC tissues (**right**). Images were downloaded from the Human Protein Atlas (HPA) (http://www.proteinatlas.org/).

Following this, we tried to characterize KRT8 protein expression in normal tissues and in lung cancer tissues by analyzing IHC staining images from the HPA database [24] and found that normal tissues had no KRT8 staining (Figure 2, left). Conversely, LUAD and LUSC had moderate-to-strong KRT8 staining (Figure 2, middle and right).

### 3.2. KRT8 Increased Expression Was Correlated with Poor OS & RFS in LUAD Patients, but not in LUSC

**Figure 3:**
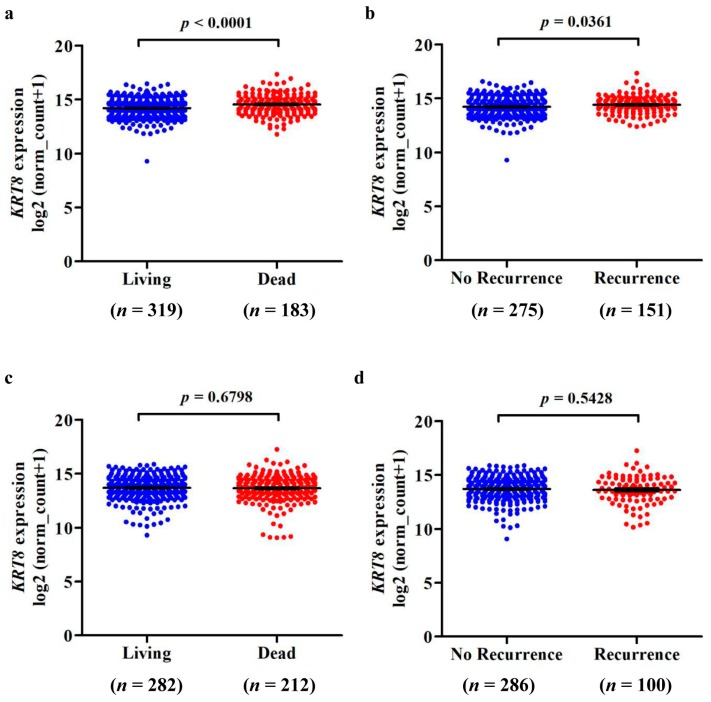
Comparison of *KRT8* mRNA expression in LUAD and LUSC patients with different survival outcomes. Comparison of *KRT8* expression in LUAD (**a**,**b**) and LUSC (**c**,**d**) patients, according to living status (**a**,**c**) and recurrence status (**b**,**d**).

To further assess the *KRT8* expression profile in different pathologic stages, we downloaded data of this kind for both LUAD and LUSC. Results showed that there was no significant difference between stages I and II, between stages II and III, and between stages III and IV, for both LUAD and LUSC (Figure S2). Following this, we compared *KRT8* mRNA expression in patients with different survival outcomes. For LUAD, we discovered that the deceased cases (*n* = 183) had significantly higher *KRT8* expression in comparison with the living cases (*n* = 319; *p* < 0.0001, Figure 3a). In addition, patients with recurrence (*n* = 151) also had increased *KRT8* expression compared to patients without recurrence (*n* = 275; *p* = 0.0361, Figure 3b). However, these associations were not found in LUSC patients (Figure 3c,d).

**Figure 4:**
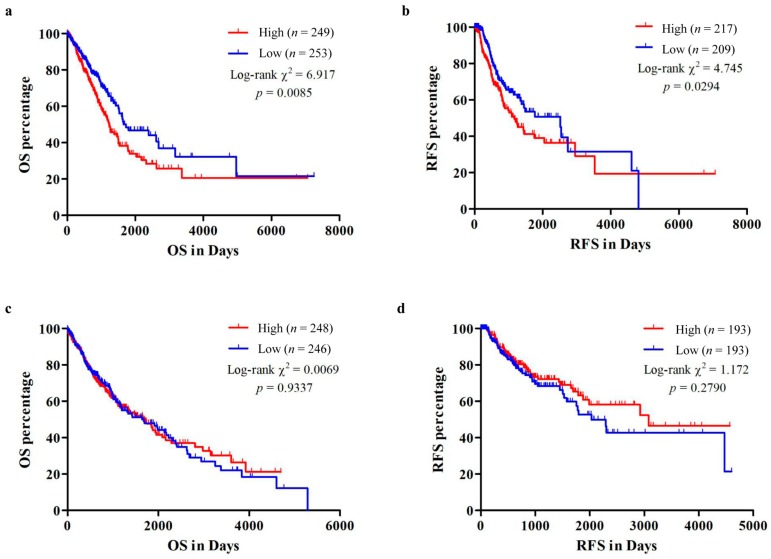
Kaplan-Meier curves of overall survival (OS) and recurrence-free survival (RFS) in LUAD and LUSC. Kaplan-Meier curves of OS (**a**,**c**) and RFS (**b**,**d**) in LUAD (**a**,**b**) and LUSC (**c**,**d**) patients.

By mapping Kaplan-Meier curves of OS and RFS, we found that LUAD patients with high *KRT8* expression had inferior OS (*p* = 0.0085) and RFS (*p* = 0.0294) in comparison to patients who had low *KRT8* expression (Figure 4a,b). In comparison, these associations were not present in LUSC patients (*p* = 0.9337 and 0.2790, respectively, Figure 4c,d). To validate these associations, we also performed data mining in the Kaplan-Meier plotter. Results showed that the high *KRT8* expression group had inferior OS (HR: 1.29; 95% CI: 1.02–1.63; *p* = 0.031) for LUAD (Figure S3a), but not for LUSC (Figure S3b). Many new treatment strategies have been developed in the past decade and these treatment strategies have had a strong effect on the RFS and OS of LUAD patients. For instance, LUAD patients with or without epidermal growth factor receptor (*EGFR*) mutation or anaplastic lymphoma kinase (*ALK*) or c-ros oncogene 1 (*ROS-1*) rearrangement had different outcomes whether they received tyrosine kinase inhibitors (TKIs) or not. Therefore, to minimize bias for patients harboring *EGFR* mutation or *ALK* or *ROS-1*, we excluded these types of patients (31, four, and three patients with *EGFR* mutation, *ALK* rearrangement, and *ROS-1* rearrangement, respectively) [30] and mapped Kaplan-Meier curves of OS and RFS for the remaining LUAD patients by setting the median *KRT8* mRNA expression. These results showed that LUAD patients with high *KRT8* expression also had worse OS and RFS (Figure S4). 

### 3.3. KRT8 Expression Was an Independent Prognostic Biomarker for Poor OS and RFS in LUAD

Consequently, we determined the independent prognostic value of *KRT8* in LUAD. The association between *KRT8* expression and the clinicopathological parameters of LUAD patients is shown in Table 1. Results show that the high *KRT8* expression group had a significantly higher ratio of patients in advanced stages (III/IV) (63/187 vs. 43/200; *p* = 0.048), male gender (128/125 vs. 103/146; *p* = 0.040), and death (108/145 vs. 75/174; *p* = 0.004) compared to the low *KRT8* expression group. By performing univariate analysis, we found that advanced stages and increased *KRT8* expression correlated with inferior OS and RFS for LUAD (Table 2). The following multivariate analysis confirmed that increased *KRT8* expression was an independent prognostic indicator in terms of OS (HR: 1.416; 95% CI: 1.050–1.909; *p* = 0.022; Table 2) and RFS (HR: 1.512; 95% CI: 1.077–2.122; *p* = 0.017; Table 2). However, there was no significant association between *KRT8* expression and OS/RFS in LUSC patients (*p* = 0.934 and 0.280 respectively, Table S1).

### 3.4. KRT8 mRNA Expression Was Regulated by Its DNA CNAs

**Figure 5:**
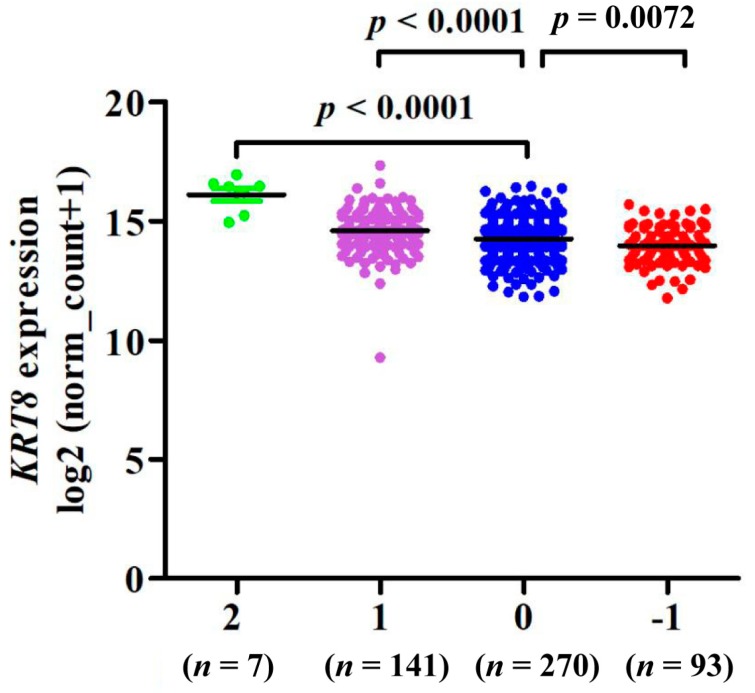
*KRT8* expression was modulated by its DNA copy number alterations (CNAs).

By analyzing *KRT8* DNA CNAs in 511 cases of LUAD, we found that although low copy gain (+1 and +2, *n* = 148, 28.96%) was not frequent, it was still associated with significantly increased *KRT8* expression compared with the copy-neutral (0) cases (Figure 5). In comparison, 93 cases (18.2%) had low-level copy loss (−1), which resulted in significantly decreased *KRT8* expression (Figure 5).

### 3.5. The Role of KRT8 in LUAD Therapies

In addition, we used three treatment-related GEO microarray datasets to explore the possible role of *KRT8* in the therapeutic response of LUAD patients. In the GSE21656 dataset [27], we found that when compared with the parental H460 cells, *KRT8* was significantly up-regulated in the cisplatin-resistant cell line (CDDP-R) (*p* = 0.0331) (Figure S5a). In the GSE6400 dataset [28], we found that *KRT8* was significantly up-regulated in the A549 cell line treated with 1.25 μM and 2.5 μM of the anti-cancer agent sapphyrin PCI-2050 (*p* = 0.0006 and *p* = 0.0005, respectively) (Figure S5b). Additionally, from the GSE6914 dataset [29], *KRT8* mRNA expression was significantly increased in the gemcitabine resistant Calu3 cell line treated with bexarotene, gemcitabine, or a two-drug combination (*p* = 0.0046, *p* = 0.0031, and *p* = 0.0328, respectively) (Figure S5c). These results show that aberrant *KRT8* expression levels might be involved in LUAD cancer treatment.

## 4. Discussion

Recently, several promising and potential prognostic biomarkers have been identified in LUAD and LUSC by secondary analysis of the TCGA data. For instance, Zhou et al. have found that epithelial cell transforming 2 (*ECT2*) expression is increased in LUAD and may predict poor OS and RFS for LUAD patients, but not for LUSC patients [31]. Using the UCSC Xena browser to analyze the same TCGA-LUAD dataset [32], Chen et al. and Yu et al. have demonstrated that high expression of lactate dehydrogenase-A (*LDHA*) [33] and the S100 protein family member *S100A16* [34] might be independent prognostic markers of inferior OS for LUAD patients. 

KRT8, predominantly expressed in epithelial cells, and its aberrant expression in multiple types of tumors is associated with cell migration [35], cell adhesion [36], and drug resistance [37]. Nevertheless, little has been reported about the expression and potential prognostic functions of *KRT8* in LUAD and LUSC. In this study, using the RNA-seq data from TCGA and protein data from the HPA, we found that when in comparison to normal lung tissues, *KRT8* expression was significantly up-regulated in both LUAD and LUSC tissues. Oncomine analysis of lung cancer versus normal tissue also confirmed that *KRT8* was significantly overexpressed in LUAD (Table 3). 

In addition, we observed that *KRT8* upregulation was associated with late clinical stages and higher ratios of recurrence and death (Figure 3). More importantly, we found that patients with high *KRT8* expression had worse OS and RFS by setting a median *KRT8* mRNA expression. Additionally, by performing univariate and multivariate analysis, we discovered that high *KRT8* expression was an independent prognostic indicator of poor OS (HR: 1.416, 95% CI: 1.050–1.909, *p* < 0.022) and RFS (HR: 1.512, 95% CI: 1.077–2.122, *p* < 0.017) for LUAD. On the contrary, although we found that *KRT8* was up-regulated in LUSC, its expression had no prognostic value in terms of OS and RFS. These results indicate that KRT8 might be a specific and promising prognostic biomarker for LUAD patients. A recent study showed that *KRT8* over-expression led to resistance to cadmium-induced adaptation and carcinogenesis [18]. KRT8 can mediate resistance to apoptosis in granulosa cell tumors by intervening in cell surface death receptor Fas (FAS) expression [43]. These studies may help to clarify the correlation between *KRT8* expression and inferior overall survival for LUAD patients.

In non-small cell lung cancer (NSCLC), the potential mechanisms of abnormal gene expression are quite complex. Genetic alterations regulating gene expression are frequently common in LUAD and have significant impacts on tumor phenotypes and patients’ survival. For instance, de novo *ERBB2* amplification confers intrinsic resistance to erlotinib in EGFR-L858R mutated TKI-naive LUAD [44]. We also explored possible clues to *KRT8* dysregulation and found that DNA low copy gain might contribute to increased *KRT8* expression in LUAD (Figure 5). Therefore, it would be worthwhile to exploring the mechanism for genetic alterations of *KRT8* influencing LUAD cell behaviors in the future. In 2017, Ricciardelli et al. found that *KRT5* mRNA expression was consistently higher in chemotherapy-resistant cells compared to chemotherapy-sensitive primary serous ovarian cancer cells and that the number of serous ovarian carcinomas with high KRT5/KRT6 or high KRT5 protein expression significantly increased following carboplatin chemotherapy [45]. Recent research has shown that KRT8/KRT18 protein levels are markedly increased in TNF-related apoptosis inducing ligand (TRAIL)-resistant cells compared to TRAIL-sensitive breast cancer cells, and they may limit TRAIL-induced apoptosis signaling via negatively regulating death receptors’ (DR5) protein stability and surface expression [46]. Through analysis of datasets obtained from the GEO database, significantly high mRNA levels of *KRT* were identified in cisplatin-resistant ADC cells. Moreover, *KRT8* was significantly increased in the ADC cell line A549 treated with sapphyrin PCI-2050, and in the gemcitabine resistant Calu3 cell line treated with bexarotene, gemcitabine, or a two-drug combination. This implies that *KRT8* may be a potential therapeutic target for LUAD. However, it is worth noting that we have only demonstrated the relationship between the prognosis of LUAD and expression of *KRT8* mRNA, but not KRT8 protein expression. In addition, knowledge of the detailed biological function of the *KRT8* gene in LUAD is lacking. Hence, the prognosis values of the KRT8 protein need further exploration, and further intensive in vitro and in vivo investigations will help clarify the underlying mechanism of *KRT8* within the pathogenesis and development of LUAD.

## 5. Conclusions

Although *KRT8* is significantly increased with regards to both RNA and protein levels in LUAD and LUSC compared to normal tissues, its expression might only serve as an independent prognostic indicator of inferior OS and RFS in LUAD and not in LUSC. 

## Figures and Tables

**Table 1:** The association between *KRT8* expression and the demographic and clinicopathological parameters of patients with primary LUAD in the Cancer Genome Atlas (TCGA).

Parameters		*KRT8* Expression	*KRT8* Expression	χ^2^	*p*-Value
		High (*n* = 253)	Low (*n* = 249)		
Age (Mean ± SD)		64.56 ± 10.11	66.10 ± 9.74		0.08
Gender	Female	125	146	4.301	0.04
	Male	128	103		
Smoking history	1	31	41	1.629	0.251
	2/3/4/5	213	203		
	Null	9	5		
Clinical stage	I/II	187	200	4.112	0.048
	III/IV	63	43		
	Discrepancy + null	3	5		
Recurrence status	No	125	150	3.056	0.086
	Yes	82	69		
	Null	46	24		
Living status	Living	145	174	8.566	0.004
	Dead	108	75		

Smoking history: 1. lifelong non-smoker; 2. current smoker; 3. current reformed smoker (for >15 years); 4. current reformed smoker (for ≤ 15 years); 5. current reformed smoker (duration not specified); Null. no data.

**Table 2:** Univariate and multivariate analysis of OS and RFS in patients with primary LUAD.

Parameters	Univariate Analysis				Multivariate Analysis			
	*p*	HR	95% Cl (Lower/Upper)		*p*	HR	95% Cl (Lower/Upper)	
OS								
Age >65 vs. ≤65	0.210	1.207	0.899	1.620				
Female vs. Male	0.670	0.939	0.702	1.256				
Smoking history 2/3/4/5 vs. 1	0.684	0.918	0.608	1.386				
Clinical stage III/IV vs. I/II	<0.001	2.651	1.945	3.612	<0.001	2.535	1.856	3.461
KRT8 expression High vs. Low	0.004	1.540	1.147	2.068	0.022	1.416	1.050	1.909
RFS								
Age >65 vs. ≤65	0.089	1.330	0.957	1.849	0.023	1.482	1.056	2.079
Female vs. Male	0.574	1.097	0.794	1.516				
Smoking history 2/3/4/5 vs. 1	0.435	1.207	0.752	1.939				
Clinical stage III/IV vs. I/II	0.006	1.699	1.160	2.489	0.021	1.580	1.070	2.333
*KRT8* expression High vs. Low	0.03	1.428	1.035	1.972	0.017	1.512	1.077	2.122

**Table 3:** Datasets of *KRT8* gene expression in LUAD.

Gene	Dataset	Normal (Cases)	Tumor (Cases)	Fold Change	*t*-Test	*p*-Value
	Selamat [13]	Lung (58)	LUAD (58)	3.846	14.527	7.59 × 10^−26^
	Landi [38]	Lung (49)	LUAD (58)	2.538	9.991	3.29 × 10^−17^
*KRT8*	Beer [39]	Lung (10)	LUAD (86)	2.183	5.838	8.30 × 10^−6^
	Su [40]	Lung (30)	LUAD (27)	2.100	4.126	8.56 × 10^−5^
	Hou [41]	Lung (65)	LUAD (45)	2.144	6.904	8.77 × 10^−10^
	Okayama [42]	Lung (20)	LUAD (226)	2.676	9.060	3.65 × 10^−10^

## Supplementary Materials

The following are available online at http://www.mdpi.com/2073-4425/10/1/36/s1: Figure S1: Heatmap of *KRT8* expression in LUAD, LUSC, and their corresponding normal lung tissues; Figure S2: Comparison of *KRT8* expression in LUAD (a) and LUSC (b) patients, according to pathologic stages; and Figure S3: The association between *KRT8* expression and overall survival for LUAD and LUSC. Kaplan-Meier curves of OS in LUAD (a) and LUSC (b) patients. Results were generated using a Kaplan-Meier plotter; Figure S4: Kaplan-Meier curves of overall survival and recurrence-free survival in LUAD patients without *EGFR* mutation or *ALK* or *ROS-1* rearrangement; Figure S5: The influence of *KRT8* on the therapeutic responses of LUAD patients. Three treatment-related microarray datasets (GSE21656, GSE6400, and GSE6914) from the GEO database were used to assess the potential roles of *KRT8* expression on therapeutic effects for LUAD (a, b, and c); Table S1: Univariate and multivariate analysis of OS and RFS in patients with primary LUSC.
